# DisProt: intrinsic protein disorder annotation in 2020

**DOI:** 10.1093/nar/gkz975

**Published:** 2019-11-12

**Authors:** András Hatos, Borbála Hajdu-Soltész, Alexander M Monzon, Nicolas Palopoli, Lucía Álvarez, Burcu Aykac-Fas, Claudio Bassot, Guillermo I Benítez, Martina Bevilacqua, Anastasia Chasapi, Lucia Chemes, Norman E Davey, Radoslav Davidović, A Keith Dunker, Arne Elofsson, Julien Gobeill, Nicolás S González Foutel, Govindarajan Sudha, Mainak Guharoy, Tamas Horvath, Valentin Iglesias, Andrey V Kajava, Orsolya P Kovacs, John Lamb, Matteo Lambrughi, Tamas Lazar, Jeremy Y Leclercq, Emanuela Leonardi, Sandra Macedo-Ribeiro, Mauricio Macossay-Castillo, Emiliano Maiani, José A Manso, Cristina Marino-Buslje, Elizabeth Martínez-Pérez, Bálint Mészáros, Ivan Mičetić, Giovanni Minervini, Nikoletta Murvai, Marco Necci, Christos A Ouzounis, Mátyás Pajkos, Lisanna Paladin, Rita Pancsa, Elena Papaleo, Gustavo Parisi, Emilie Pasche, Pedro J Barbosa Pereira, Vasilis J Promponas, Jordi Pujols, Federica Quaglia, Patrick Ruch, Marco Salvatore, Eva Schad, Beata Szabo, Tamás Szaniszló, Stella Tamana, Agnes Tantos, Nevena Veljkovic, Salvador Ventura, Wim Vranken, Zsuzsanna Dosztányi, Peter Tompa, Silvio C E Tosatto, Damiano Piovesan

**Affiliations:** 1 Department of Biomedical Sciences, University of Padova, Padova 35121, Italy; 2 MTA-ELTE Lendület Bioinformatics Research Group, Department of Biochemistry, Eötvös Loránd University, Budapest 1117, Hungary; 3 Departamento de Ciencia y Tecnología, Universidad Nacional de Quilmes - CONICET, Bernal, Buenos Aires B1876BXD, Argentina; 4 Consejo Nacional de Investigaciones Científicas y Técnicas. Instituto de Investigaciones Biotecnológicas IIBIO, Universidad Nacional de San Martín, San Martín, Buenos Aires, Argentina; 5 Computational Biology Laboratory, Danish Cancer Society Research Center, Copenhagen DK-2100, Denmark; 6 Department of Biochemistry and Biophysics and Science for Life Laboratory, Stockholm University, Box 1031, Solna 17121, Sweden; 7 Biological Computation & Process Laboratory, Chemical Process & Energy Resources Institute, Centre for Research & Technology Hellas, Thessalonica GR-57500, Greece; 8 Departamento de Fisiología y Biología Molecular y Celular (DFBMC), Facultad de Ciencias Exactas y Naturales, Universidad de Buenos Aires, Buenos Aires, Argentina; 9 Division of Cancer Biology, The Institute of Cancer Research, Chelsea, London SW3 6BJ, UK; 10 Laboratory for Bioinformatics and Computational Chemistry, Institute of Nuclear Sciences Vinca, University of Belgrade, Belgrade 11001, Serbia; 11 Center for Computational Biology and Bioinformatics, Indiana University School of Medicine, IN 46202, USA; 12 Swiss Institute of Bioinformatics and HES-SO \ HEG, Geneva 1200, Switzerland; 13 Structural Biology Brussels, Vrije Universiteit Brussel (VUB), Brussels 1050, Belgium; 14 VIB-VUB Center for Structural Biology, Flanders Institute for Biotechnology (VIB), Brussels 1050, Belgium; 15 Institute of Enzymology, Research Centre for Natural Sciences, Hungarian Academy of Sciences, Budapest H-1117, Hungary; 16 Departament de Bioquímica i Biologia Molecular and Institut de Biotecnologia i Biomedicina, Universitat Autònoma de Barcelona, Bellaterra 08193, Spain; 17 Centre de Recherche en Biologie cellulaire de Montpellier (CRBM), UMR 5237 CNRS, Université Montpellier, Montpellier 34293, France; 18 Institut de Biologie Computationnelle(IBC), Montpellier 34095, France; 19 Department of Woman and Child Health, University of Padova, Padova 35127, Italy; 20 Fondazione Istituto di Ricerca Pediatrica (IRP), Città della Speranza, Padova 35127, Italy; 21 Instituto de Biologia Molecular e Celular (IBMC) and Instituto de Investigação e Inovação em Saúde (i3S), Universidade do Porto, Porto 4200-135, Portugal; 22 Bioinformatics Unit. Fundación Instituto Leloir, Ciudad de Buenos Aires C1405BWE, Argentina; 23 Translational Disease Systems Biology, Faculty of Health and Medical Sciences, Novo Nordisk Foundation Center for Protein Research University of Copenhagen, Copenhagen DK-2200, Denmark; 24 Bioinformatics Research Laboratory, Department of Biological Sciences, University of Cyprus, Nicosia, CY 1678, Cyprus; 25 Interuniversity Institute of Bioinformatics in Brussels (IB2), ULB-VUB, Brussels 1050, Belgium; 26 CNR Institute of Neurosceince, Padova 35121, Italy

## Abstract

The Database of Protein Disorder (DisProt, URL: https://disprot.org) provides manually curated annotations of intrinsically disordered proteins from the literature. Here we report recent developments with DisProt (version 8), including the doubling of protein entries, a new disorder ontology, improvements of the annotation format and a completely new website. The website includes a redesigned graphical interface, a better search engine, a clearer API for programmatic access and a new annotation interface that integrates text mining technologies. The new entry format provides a greater flexibility, simplifies maintenance and allows the capture of more information from the literature. The new disorder ontology has been formalized and made interoperable by adopting the OWL format, as well as its structure and term definitions have been improved. The new annotation interface has made the curation process faster and more effective. We recently showed that new DisProt annotations can be effectively used to train and validate disorder predictors. We believe the growth of DisProt will accelerate, contributing to the improvement of function and disorder predictors and therefore to illuminate the ‘dark’ proteome.

## INTRODUCTION

About 20 years ago, the concept of the intrinsic structural disorder of proteins came into being (1,2). Since then, the field has reached adulthood, with the concept of protein disorder gaining wide acceptance in the community. Intrinsically disordered proteins/regions (IDPs/IDRs) are now often being referred to without a citation, the term having become as common as the ‘globular’ structure of a protein, or the ‘active site’ of an enzyme. Yet, the field is still accelerating and has not reached its climax, as signaled by several recent breakthroughs and high-impact stories (3,4).

For example, it was recently recognized by ‘omics’ data analyses that about half of eukaryotic proteins are ‘dark’, in the sense that we have no information on their 3D structure (5), which poses a serious bottleneck in their functional characterization and annotation. Similarly, only 45% of the residues of all human proteins are covered by multiple sequence alignment-based Pfam-A protein family annotations (6). These values suggest that we have only a vague notion about the structure and function of the majority of proteins in our databases. As a significant fraction of the dark proteome and non-Pfam annotated proteins and protein regions are intrinsically disordered (the concepts having become almost synonymous), our best approach for illuminating the dark proteome is to predict disorder from sequence, and experimentally characterize the underlying structural ensembles (7).

The prediction of protein disorder from sequence was on the menu of the Critical Assessment of Protein Structure Prediction (CASP), a community-wide experiment of predicting protein structures from sequence (8), for many years. A new initiative, the Critical Assessment of Intrinsic protein Disorder (CAID), has now reached maturity and will be reintegrated into the CASP programme, with a clearer IDP perspective. New annotations in DisProt have already been used to provide a blind evaluation of disorder predictors (9).

Several recent breakthroughs have also signaled the vitality of the field. An unsettled question with IDPs/IDRs is whether their structural disorder persits in the crowded interior of cells. Whereas diverse indirect evidence indicates that this is the case (10), only in-cell NMR seems currently available to address this issue. For example, it was recently applied to study Parkinson's disease protein α-synuclein (DisProt DP00070), once suggested to have folded, oligomeric structure in cells (11). In-cell NMR has clearly shown that α-synuclein preserves its disordered, monomeric state in non-neuronal and neuronal cells alike (12).

Another aspect of the functionality of IDPs is that they often mediate protein-protein interactions, mostly by folding upon partner binding (13), but sometimes by preserving their structural disorder (fuzziness) in the bound state (14). This was recently shown to occur in the extremely tight (picomolar) interaction between two human IDPs, histone H1 (DisProt DP01156) and its nuclear chaperone, prothymosin-α (DisProt DP01677). These proteins associate while retaining their highly dynamic, fully disordered state (15). Functional regulation of another type may also arise from structural disorder, via the entropic force generated by the structural ensemble of an IDP/IDR. In the enzyme UDP-α-D-glucose-6-dehydrogenase (UGDH, DisProt DP02338), the C-terminal disordered tail has such a role, fine-tuning the energy landscape of the protein and stabilizing a sub-state that has a high affinity for an allosteric inhibitor (16,17).

It is without doubt that we cannot afford to ignore this intrinsically disordered, yet functionally important part of the proteome. Not only does structural disorder play an exquisite role in cellular signaling and regulation (18), it is also often implicated in disease (19,20). Consequently, IDPs also represent important drug targets: a largely unexplored frontier in developing molecular medicine is the rational design of drugs against IDPs (21,22).

Due to these challenges, it is important to update and upgrade DisProt, the primary database of protein disorder. Whereas predicted disorder features are available in MobiDB (18), which has recently been integrated in UniProtKB (23), the crux of understanding protein disorder is the availability of manually curated, experimentally verified disorder annotations. The previous release of the database, DisProt 7 (24), held data of ∼800 entries of IDPs/IDRs. Other databases, like IDEAL (22), ELM (25), DIBS (26) and MFIB (27), also include curated disorder information but are somehow different capturing specific functional aspects, or protein classes, and the overlap with DisProt is minimal (28). To reflect on the above-noted breakthroughs and the recent explosion of the related liquid-liquid phase separation (LLPS) field (29), we present a significant update and upgrade of the DisProt database, which is now at version 8. DisProt 8 holds almost two-times as many entries as DisProt 7, including the majority of those available in aforementioned databases.

DisProt has been completely redesigned with an extended and updated functional classification scheme that relies on functional/structural aspects of annotated regions and incorporates a novel functional class ‘biological condensation’. Annotation concepts have been formalized in a new Disorder Ontology (DO), which is maintained by the entire DisProt community.

DisProt 8 also has many novel features that make it easier to search. The graphical interface has been redesigned and a new entry format provides greater flexibility, simplifies maintenance and allows the capture of more information from the literature.

Lastly, we made significant improvements on the new annotation interface used by DisProt curators to populate the database. It is now easier to use and leverages curators’ work by enabling text-mining technologies, integrating third-party information on-the-fly and implementing several validation checks.

In recent work, specific sequence features have been associated with different disorder ‘flavours’ and mapped on a large scale (30). This information has been used to improve protein function prediction from sequence (31). We believe the growth of DisProt will accelerate, contributing to the improvement of function and disorder predictors and therefore to illuminate the ‘dark’ proteome.

## PROGRESS AND NEW FEATURES

### Database structure and implementation

The way disorder information is represented in the literature is inherently complex. Articles describe functional and structural aspects, where IDPs are strictly connected to dynamic behavior. DisProt tries to capture as much biological knowledge as possible while at the same time providing simple and clear annotations. The idea is to optimize user experience and improve data exchange with other major annotation resources.

### Database records

The major change compared to the previous release is the new annotation paradigm. In DisProt 7, experimental methods represented the annotation core of a DisProt region and function terms were used as attributes. Now the core of an annotation is the functional/structural aspect of a region and the experimental method is an attribute representing the quality of the annotation. Both functional/structural aspects and the type of evidence are encoded in a controlled vocabulary, in line with other core data resources (e.g. UniProtKB). In the new DisProt region format, a ‘statement’ field has been introduced to track the literature text supporting the evidence. When the text is too long or complicated, a curator statement is provided instead. All ‘statements’ are available from the website and could be used to train text-mining algorithms and to highlight sentence-based annotations on abstracts and full text articles. A new ‘obsolete’ field has been introduced in order to track regions which have been excluded from the current release. It also includes the reason for obsolescence, usually changes in the reference sequence due to UniProKB updates or curator errors.

At present, functional terms can be associated to a subset of disordered residues, i.e. to a region shorter than the one for which disorder has been experimentally evaluated. For example, a paper describing a folding upon binding event can provide two DisProt records, one region spanning the folding residues and another showing the interacting ones. All regions have now a region identifier field which is unique and stable, i.e. it is never reused and becomes obsolete if the reference sequence changes. Functional and structural vocabulary terms along with experimental methods have been encoded in a new Disorder Ontology (DO).

### Disorder ontology

In order to describe the different functional aspects of IDPs and the experimental methods used to characterize them, an annotation scheme was introduced in DisProt 7. A more formalized version of the disorder ontology was implemented in DisProt 8, to move towards a descriptive, interoperable and collaborative ontology of IDPs. This is the first release of the Disorder Ontology in the specific Biomedical Ontology (OBO) or the Web Ontology Language (OWL) formats (32,33). Besides improving the ability to reuse and share the ontology, these formats allow definition of label attributes such as ‘xterm’ (cross-references to external databases or ontologies) and ‘synonym EXACT’ (alternative names). They also support assignment of relationships among terms (including for example ‘disjoint_from’ to mark terms that should not be linked together).

An identifier was assigned to each term in the ontology. It gives each label an 8-character accession code (e.g. ‘DO:00001’), with the string ‘DO:’ to indicate the disorder ontology and five numeric characters to indicate the term unambiguously. Mirroring the Gene Ontology, accession numbers are assigned incrementally and there is no relationship between accession codes and the ontology topology.

We have reviewed the terms and organization of the whole ontology, paying particular attention to the ‘Function’ category. We made some straightforward changes, for example, we split ‘Fatty acylation (myristoylation and palmitylation)’ into a renamed parent class ‘Fatty acylation’ and its new children terms ‘Myristoylation’ and ‘Palmitoylation’. A new functional term was also introduced to annotate different phenomena related to ‘Biological condensation’ (DO:00040). It describes proteins that undergo phase separation from a solution, e.g. either to form a dynamic liquid droplet (DO:00041, ‘liquid–liquid phase separation’) or a hydrogel (DO:00042). It also includes cellular protein condensates (DO:00045 and DO:00046 describe ‘granule’ and ‘cellular puncta’, respectively), regardless of their existence in physiological or pathological states (as in ‘Amyloid’, DO:00046). This class provides an initial scheme to annotate the relevant but still scarce information available about protein condensates, and we expect this subset of the hierarchy to be modified (possibly by conforming its own sub-ontology) as the field matures.

The distinction between structural states and dynamic events, like disorder-to-order transitions, has been made clearer. Previously ‘Structural state’ terms were part of the ‘structural transition’ category and ‘disorder’ was only used implicitly. Now, a new ‘structural state’ category has been created and it includes ‘disorder’, ‘order’, ‘pre-molten globule’ and ‘molten globule’ terms. In the future, structural states will be annotated in conjunction with the corresponding environmental conditions affecting the conformation (pH, post-translational modifications (PTMs), temperature, etc.).

All experimental methods are now encoded under the ‘detection method’ branch. An overlap with other ontologies exists, but it is not complete or the definition of the same experiment is often slightly different. For example, in DisProt the term ‘crystallography’ includes ‘missing electron density’ as a child. In other ontologies ‘crystallography’ always indicates methods for structural determination. A new ‘electron cryomicroscopy’ (DO:00128) term has been also introduced in DisProt 8.

The Disorder Ontology (version 0.1.0) is maintained by the DisProt consortium and is available to be adopted by other databases for general use. In the future, it will be made available also from third party dedicated repositories.

### Curation process and updates

DisProt data is provided by a community effort and annotations are collected through a web interface, which has been improved drastically compared to the previous version in terms of field validation, autocompletion and Named Entity Recognition (NER). In particular, curators can use a dedicated service from the NextA^5^ literature triage infrastructure (34) to rank relevant literature starting from a gene name. In complement, when curators start from an article, the DisProt interface exploits the SciLite software through the EuropePMC API (35) to automatically retrieve biological entities and identifiers in the manuscript.

The annotation interface implements the concept of ownership and user privileges. DisProt distinguishes two types of users, curators and reviewers. Curators can edit only entries that they have created, while reviewers can modify all entries. Before release, the reviewers check all annotations to ensure high quality of the data. Curators are experts in the field and trained to meet DisProt annotation standards. As a community database, DisProt looks for new curators. Curator candidates are enrolled upon an evaluation of the curriculum and curation skills.

Access to the annotation interface is restricted to registered curators and provided through Google Authentication (based on the OAuth 2.0 protocol) or the ELIXIR authentication and authorization infrastructure system (36). In the past, the DisProt interface had been kept open for limited time slots. Now the new DisProt interface is always open and new releases will be delivered more frequently, i.e. every six months.

DisProt versioning has been improved. A numeric identifier indicates the version of the database entry, e.g., version ‘8.0’ and a ‘<year>_<month>’ code indicates the version (timestamp) of annotated data, e.g. ‘2019_09’.

### Database content

Since the last release, both the number of proteins and regions has almost doubled. DisProt 8 contains 1556 proteins and 3511 sequence segments annotated as disordered, which cover 19.7% of the number of residues. These numbers become 1390 proteins, 3041 regions and 18.7% of disorder content when ambiguous evidence is not considered. Previous annotations have been fixed and updated. Regions shorter than ten residues are no longer allowed and existing short regions were marked as obsolete as the majority are flexible loops annotated from X-ray experiments that do not represent disorder-related functional sites. Regions ending outside the sequence, regions with a start index of zero instead of one and entries for which the reference sequence in UniProtKB changed, were corrected and, when necessary, new records were created manually.

**Figure 1.**
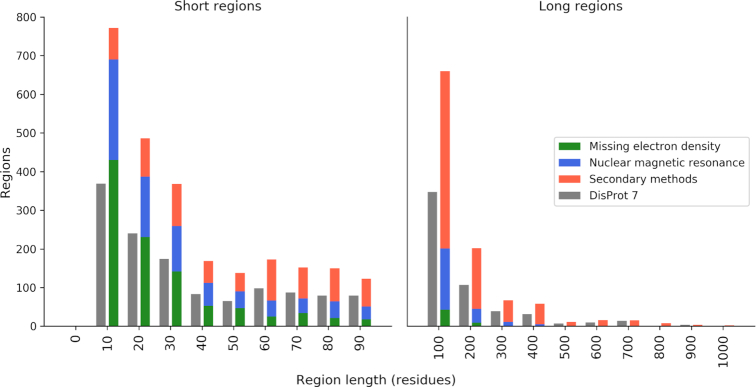
Distribution of region length. Regions shorter than 100 residues (left) are binned in groups of 10 residues. Regions longer than 100 (right) are binned in 100 residues. The tick labels indicate the lower bound which is included. Gray bars refer to the previous release (DisProt 7).

Figure 1 shows the distribution of regions based on their length and experimental detection method. Compared to the previous version, the distribution shape has not changed. Secondary methods, which include all ‘detection methods’ terms except ‘missing electron density’ (DO:00130) and ‘nuclear magnetic resonance’ (DO:00120) dominate experiments used to identify longer (>100 residues) regions.

**Figure 2.**
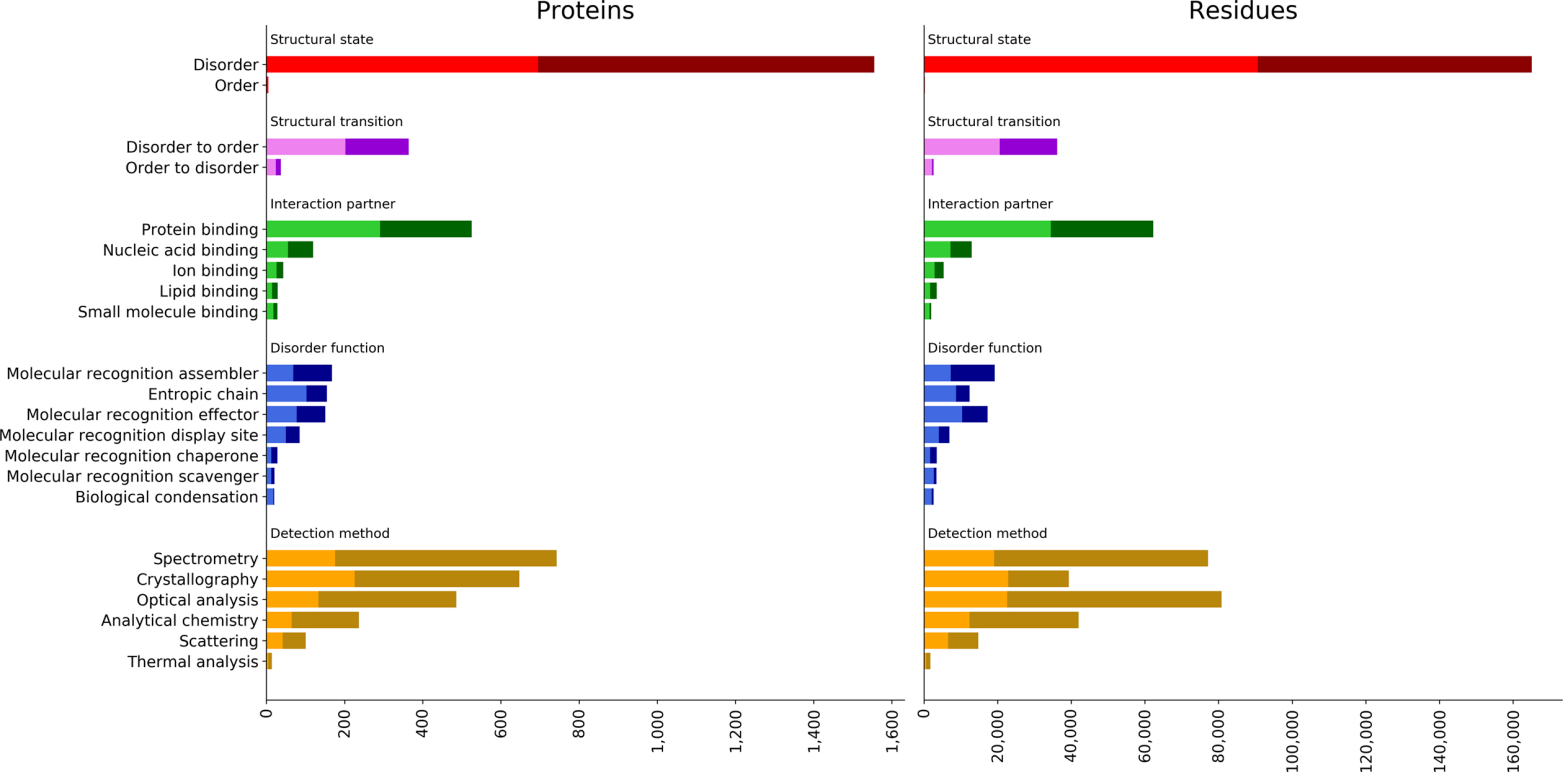
Distribution of disorder annotation terms. Terms belong to the Disorder Ontology and only those one node away from the ontology root are shown. Annotation counts for child terms are propagated to parents up to the root. The dark segments correspond to proteins (left) or residues (right) for which more than one piece of evidence is available. Different ontology aspects (namespaces) are grouped and have different colors.

The statistics on annotation data for the main branches of the disorder ontology are reported in Figure 2. Only terms one node away from the ontology root are considered and more specific annotations are propagated following the ‘true path rule’, i.e. following the ontology hierarchy, so that parent terms account for children counts.

Different ontology aspects (‘namespace’ field in DisProt records), are shown with different colors. In red the ‘structural state’ terms show as the majority of region records in DisProt are annotated as disordered. Only five proteins are annotated with the ‘order’ term. In the future, curators will be encouraged to also track information about order, in particular when relevant for structural transitions. Transitions are mainly covering folding events (‘disorder to order’), 365 proteins and 36 200 residues, and not the contrary. The majority of interaction partner annotations refers protein and nucleic acid binding. Binding residues are, however, overestimated since in the previous DisProt version, due to hard constraints in the database schema, it was not possible to narrow region boundaries to real interacting positions. Binding positions will become more precise in the future. The new term introduced in DisProt 8, ‘Biological condensation’ (DO:00040) has been assigned to a total of 20 proteins, 29 regions and 2610 residues. The new ‘electron cryomicroscopy’ (DO:00128) term, which is a child of ‘crystallography’, covers 34 proteins, 67 regions and 4726 residues.

Darker segments in Figure 2 indicate the fraction of proteins (left plot) and residues (right plot) for which more than one experimental evidence is available. At the bottom in orange the distribution of ‘Detection methods’ terms. ‘Proteins’ and ‘residues’ distributions have a similar shape. ‘Crystallography’, which is a parent of ‘missing electron density’, covers less residues compared to ‘spectrometry’ and ‘optical analysis’, indicating that regions identified with crystallographic techniques are shorter on average. Moreover, ‘crystallography’ has less residues covered by multiple experimental evidence compared to other techniques. In general, disorder annotation is well supported with 44.4% of disordered proteins and 43.2% of the disordered residues backed by two or more literature references.

### DisProt website

The DisProt website has been completely redesigned, improving the user experience, visualization and functionalities. Additionally, a big effort was made to develop the DisProt Application Programming Interface (API) to enable users to retrieve a single entry or a region and to perform advanced searches via RESTful endpoints (URLs). The new API and distribution formats are extensively documented in the help page.

### Entry page

The entry page is composed of three main sections. On the top, general information of the protein including name, DisProt ID, organism, sequence length, MobiDB and UniProtKB accession numbers are provided. On the top right, it is possible to select the DisProt version and hide/show ambiguous/obsolete evidence. A download dropdown button allows saving the whole entry data in JSON, TSV (tab-separated) or the corresponding sequence in FASTA format.

A new dynamic feature viewer allows to visualize DisProt evidence mapped onto sequence. The feature viewer shows two tracks by default, DisProt consensus and domains, the latter including Pfam (37) and Gene3D (38) annotation. DisProt consensus is generated by merging region annotation following the hierarchy of the ontology terms. In the last step, when merging the four main ontology branches, priority is given to ‘interaction partner’, ‘structural transition’, ‘structural state’ and ‘disorder function’, respectively.

The feature viewer can be expanded to see sub tracks and it is possible to zoom in and out specific regions, customize the view and download a high quality image. Region tooltips are activated on mouse over and provide detailed information about the corresponding annotation.

Region details are also provided on the bottom of the page, organized in a dynamic list of boxes. A search box, which supports regular expressions, allows to filter the list of regions. The filter is also applied to the feature and sequence viewers (right) in real time, for example, by typing ‘nuclear magnetic resonance’ it is possible to select only region evidence from NMR experiments.

### Browsing and searching data

DisProt implements both a database and a BLAST search (39), both available from the ‘browse’ page. The database search allows to compose a query against several fields, which can be combined to meet multiple criteria. All search fields accept regular expressions, and ‘Free text’ allows to search against the entire database content. For example, by searching ‘p53’ in ‘free text’ and ‘homo | mus’ in ‘organism’ will return all human and mouse proteins with the ‘p53’ string somewhere in the corresponding database records (protein name, annotation reference title, etc.). Query results are displayed in the table below the search box. Table columns are customizable and the result can be downloaded in JSON, TSV or FASTA format.

### DisProt API

DisProt provides programmatic access to perform a search through REpresentational State Transfer (or RESTful) Web Service API. A single entry or evidence can be retrieved by using DisProt or UniProtKB identifiers. Additionally, a text search against the entire database can be performed by specifying query fields (name, organism, etc.) directly as URL parameters in the HTTP request. JSON, TSV and FASTA formats are supported.

## CONCLUSIONS AND FUTURE WORK

In the previous release, DisProt disorder annotations were polished and major errors were fixed but the number of newly annotated proteins was limited. In DisProt 8, disorder annotations doubled and a robust infrastructure has been put in place to leverage and accelerate the annotation process. The database format has been improved to be flexible enough to capture essential information from the literature but, at the same time, keeping disorder representation simple and clear. A new disorder ontology has been formalized with the aim of improving maintenance and data exchange with core data resources. The new ontology is versioned and provides a hierarchy to facilitate term traversal. Article sentences tracking statements about disorder experimental evidence are now captured providing a corpus for the implementation of new text-mining models. New protein examples are used as ground-truth to evaluate prediction methods as in the Critical Assessment of Disorder Annotation (CAID). DisProt long term sustainability is guaranteed by the centrality of DisProt in several initiatives involving large communities of bioinformaticians working on disorder, such as the IDPfun Marie Curie RISE and the ELIXIR IDP User Community.
